# Case Report: Prolonged remission of metastatic cisplatin-refractory nasopharyngeal carcinoma with Pembrolizumab

**DOI:** 10.3389/fonc.2023.1249453

**Published:** 2023-11-08

**Authors:** Wei Cen Wang, Beatrice Preti, Nancy Read, Paul Gibson, Keith Kwan, Eric Winquist

**Affiliations:** ^1^ Schulich School of Medicine and Dentistry, University of Western Ontario, London, ON, Canada; ^2^ Division of Radiation Oncology, Department of Oncology, University of Western Ontario and London Health Sciences Centre, London, ON, Canada; ^3^ Division of Hematology and Oncology, Department of Pediatrics, McMaster University Faculty of Health Sciences and McMaster Children’s Hospital, Hamilton, ON, Canada; ^4^ Department of Pathology and Laboratory Medicine, University of Western Ontario and London Health Sciences Centre, London, ON, Canada; ^5^ Division of Medical Oncology, Department of Oncology, University of Western Ontario and London Health Sciences Centre, London, ON, Canada

**Keywords:** nasopharyngeal carcinoma, PD-1 inhibitor, pembrolizumab, overall survival, progression free survival, case report

## Abstract

**Background:**

Epstein-Barr virus (EBV)-related nasopharyngeal cancer (NPC) is a common type of cancer in certain areas of the world such as southeast Asia, but is uncommon in Canada. There is currently no reliably effective standard treatment for incurable metastatic EBV-related NPC that progresses after first-line therapy with gemcitabine/cisplatin.

**Methods:**

With his consent, the health records of a patient with relapsed metastatic EBV-related NPC treated with pembrolizumab immunotherapy were retrospectively reviewed and reported.

**Case report:**

A male patient presented at age 15 with stage IVA EBV-related NPC. Despite response to initial chemoradiation and adjuvant chemotherapy, the patient experienced metastatic cancer relapse in lymph nodes and bone. There was initial response to gemcitabine/cisplatin chemotherapy, but the cancer progressed after 7 cycles. The patient was then switched to pembrolizumab and had a near complete clinical response after 14 cycles. Serum EBV titers have normalized and CT imaging shows only some healed bone metastasis. Retrospective assessment of tumor CPS PD-L1 was >20. Hypothyroidism developed, possibly due to radiation treatment, but otherwise he did not experience any other immune-mediated toxicities on or following treatment, which lasted in total 2 years with 41 cycles. To date, the patient has been observed off pembrolizumab for over one year and is highly functional without evidence of disease progression.

**Conclusion:**

This case illustrates the potential benefit of immunotherapy for improving survival and quality of life in selected patients with metastatic EBV-positive cisplatin-refractory NPC.

## 1 Introduction

Nasopharyngeal carcinoma (NPC) arises from the epithelium of the nasopharynx and has a high tendency to metastasize. The incidence of Epstein-Barr virus (EBV)-associated NPC is variable worldwide, rare in most areas, but endemic in east and southeast Asia, ranking 8th in cancer mortality (1, 2). Cure rates are high for patients presenting with localized NPC, and treatment has been changing rapidly. In addition to chemoradiation, neoadjuvant chemotherapy and maintenance capecitabine therapy have all reported overall survival (OS) benefits in randomized control trials (RCTs) (3–6). For patients with incurable recurrent or metastatic disease, gemcitabine/cisplatin chemotherapy is the standard of care; however, reliably effective second-line treatment options are lacking (7). Pembrolizumab is a monoclonal antibody that binds to programmed cell death protein 1 (PD-1) on T cells, preventing an inactivation signal from programmed death ligand 1 (PD-L1) on tumor cells, thereby enabling T cells to kill tumor cells. Pembrolizumab has shown efficacy in multiple tumor types in RCTs (8–11). However, the role of immunotherapy in recurrent/metastatic (RM) NPC is unclear. Herein with their consent, we report a case of prolonged clinical and biochemical remission with pembrolizumab in a patient with cisplatin-refractory RMNPC.

## 2 Case description

**Figure 1:**
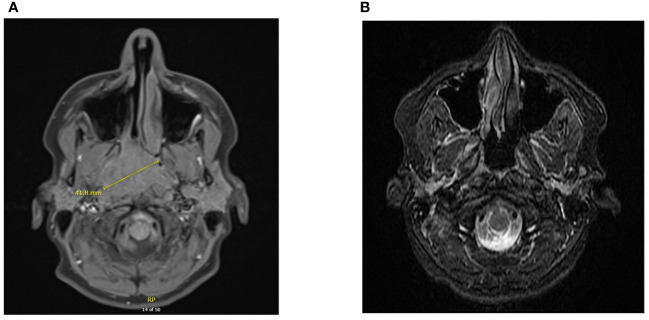
Diagnostic imaging of NPC at primary site. CT scan demonstrating a large mass at the right nasopharynx upon diagnosis **(A)**. MRI of nasopharynx demonstrating complete response following chemoradiation and adjuvant chemotherapy **(B)**.

A 15-year-old previously healthy male presented with a 6 months history of severe right nasal congestion and rhinorrhea. Computed tomography (CT) and magnetic resonance imaging (MRI) demonstrated a large right nasopharyngeal-enhancing soft tissue mass obstructing the nasopharyngeal airway and extending into the right posterior nasal cavity, pterygopalatine fossa, nasopalatine fossa and inferior sphenoid sinus (**Figure 1A**). Bilateral retropharyngeal and level II A/B adenopathy was present. Biopsy of the mass showed infiltrating mature lymphocytes and neoplastic cells strongly and diffusely positive for EBV-LMP and EBER; CPS PD-L1 was >20. The patient was diagnosed with T4N1 EBV-positive undifferentiated non-keratinizing NPC and started on 7 weeks of curative-intent radiation with concurrent chemotherapy. Radiation was given as 70 Gy in 35 fractions to the gross tumor and 56 Gy to the bilateral nodes. Intravenous (IV) cisplatin was given concurrently on days 1, 22 and 43 at 100 mg/m^2^. After completion, adjuvant chemotherapy with daily infusional fluorouracil 1000 mg/m^2^ for four days and IV cisplatin 80 mg/m^2^ was given for 3 cycles, with cisplatin omitted due to ototoxicity the last cycle.

**Figure 2:**
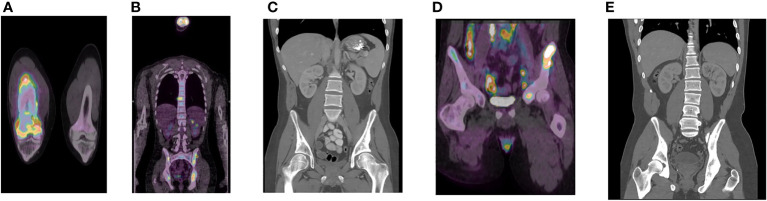
Diagnostic imaging of NPC metastasis. PET scan demonstrating bony lesions in the right femur **(A)** and multiple iliac bony lesions and metastatic adenopathy in the abdomen, retroperitoneum, and chest **(B)** prior to gemcitabine/cisplatin (G/C) chemotherapy. CT scan after 4 cycles of G/C chemotherapy demonstrating resolution of metastatic adenopathy **(C)**. PET scan after 8 cycles of G/C chemotherapy demonstrating cancer progression in the ilium, retroperitoneum, and pelvis **(D)**. Latest CT scan to date (22 months post-pembrolizumab) demonstrating stable healed bony metastasis **(E)**.

Post-treatment MRI scans suggested complete response (**Figure 1B**). He was placed on thyroid hormone replacement for post-treatment hypothyroidism. At 6 months post-adjuvant chemotherapy, the patient developed right thigh pain. Radiographs showed periosteal reaction in the distal femoral diaphysis and, as the patient was a competitive ice hockey player, this was considered likely a stress fracture. A subsequent biopsy was negative for malignancy. The discomfort persisted and worsened, with two-years post-treatment MRI scan of the femur showing worsening of aggressive bony changes and the appearance of new lesions suggestive of osteomyelitis or metastatic cancer. A PET scan performed 29 months post-treatment showed multifocal bony lesions and multiple hypermetabolic lymph nodes in the chest, abdomen and retroperitoneum without evidence of disease recurrence in the primary nasopharyngeal site (**Figures 2A, B**). Biopsy of the right femur confirmed EBV-positive metastatic carcinoma.

The patient had moderately severe chronic right thigh pain, hemoglobin was 70 g/L, and serum EBV DNA titer was 678,000 IU/ml (**Table 1**). Treatment was initiated with analgesics, packed red cell transfusion, palliative radiation 2000 Gy in 5 fractions to the femur, zoledronic acid 4 mg IV, and gemcitabine/cisplatin chemotherapy on a 21 day schedule. There was rapid improvement in the patient’s pain and a CT of the abdomen and pelvis following cycle 4 chemotherapy demonstrated complete resolution of the retroperitoneal and pelvic metastatic adenopathy (**Figure 2C**). EBV titers nadired at 4000 IU/ml at chemotherapy cycle 5, and then began to slowly increase (**Table 1**). Following cycle 7, the patient’s thigh pain recurred and he also developed progressive anemia which required transfusion. His energy level progressively deteriorated and he required wheelchair assistance. PET/CT after cycle 8 demonstrated cancer progression with new intrathoracic, retroperitoneal, and pelvic nodal lesions and left iliac bony lesion (**Figure 2D**).

**Table 1:** Patient’s PCR EBV DNA quantification throughout treatment.

Date	EBV Titer (IU/mL)
2016/06/13: adjuvant chemotherapy complete	
**2018/11/27**	6.78x10^5^
2018/12/10: gemcitabine/cisplatin initiated	
**2018/12/31**	8.45x10^4^
**2019/01/21**	6.13x10^3^
**2019/02/13**	9.44x10^3^
**2019/03/4**	4x10^3^
**2019/03/25**	1.03x10^4^
**2019/04/25**	4.97x10^4^
**2019/05/06**	5.66x10^4^
2019/05/13: pembrolizumab initiated	
**2019/06/24-present**	Undetectable/Detected, but less than lower limit of quantification

EBV, Eptein-Barr virus.

Chemotherapy was discontinued, zoledronic acid was maintained, and the patient was switched to 200 mg pembrolizumab every 21 days. Improvement in hemoglobin level were seen and the patient reported decreased pain and increased energy level after cycle 1. A CT scan following cycle 5 showed size reduction of lymph nodes in the chest, abdomen, and pelvis and stable bony metastasis in the pelvis. By cycle 6, the patient was able to engage in full range of physical activities and by cycle 10, his EBV titer was undetectable (**Table 1**). He appeared well with no signs, symptoms, or abnormal blood work suggesting disease progression, and did not experience any obvious immune-mediated toxicities. Subsequent CT results to date show only stable healed bony metastasis (**Figure 2E**).

**Figure 3:**
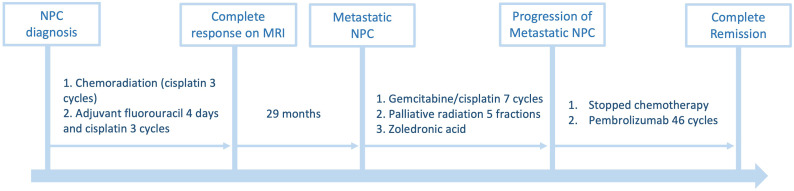
Timeline of disease progression and treatment.

Pembrolizumab was discontinued after 31 months (46 cycles). The patient is currently being surveilled quarterly with EBV serology and CT imaging, and receives maintenance zoledronic acid. Almost 2 years after discontinuing pembrolizumab, his physical examination, blood work, and CT are unremarkable, EBV levels are undetectable, and he has resumed playing competitive ice hockey. The patient’s treatment timeline and EBV titers are summarized in **Figure 3**; **Table 1**, respectively.

## 3 Discussion

EBV-related NPC is an aggressive disease differing from other mucosal head and neck tumors in its higher tendency for distant metastases with a *de novo* metastasis rate up to 15% and post-curative treatment metastasis rate up to 18% (12, 13). Despite its apparent chemosensitivity, RMNPC has been associated with a poor outcome, with median OS of approximately 20 months (14). EBV greatly contributes to the pathogenesis of EBV-positive NPC, which provides opportunities for immunotherapy. EBV-induced latent membrane protein 1 (LMP1) was shown to cooperate with interferon gamma (IFN- γ) and other pathways to upregulate PD-L1 on NPC cells, thereby escaping immune surveillance (15). Furthermore, EBER-positive RMNPCs, which indicates active EBV infection in tumor cells, were shown to have significantly better PFS and OS compared to EBER-negative RMNPCs when treated with pembrolizumab or nivolumab (16). Therefore, although plasma EBV DNA level correlated with higher rates of recurrence and metastasis and lower OS in NPC, it may be associated with better efficacy of pembrolizumab, as this drug allows the recognition of cancer cells despite PD-L1 upregulation (17, 18). Our patient had extremely high plasma EBV titers prior to initiating immunotherapy and high tumor PD-L1 expression with positive EBER, providing possible explanations for their excellent response to pembrolizumab. Nonetheless, the association between plasma EBV level, tumor PD-L1 expression, and outcomes with PD-1 inhibitors requires further characterization, as patients with higher pre-treatment EBV levels interestingly had a shorter median OS when treated with toripalimab, another PD-1 inhibitor (19). In addition to tumor markers, evidence suggest that the tumor microenvironment (TME) could also influence the response to immunotherapy in NPC. It was found the subtype of NPC TME with abundant immune cells and immune response and activated stroma was associated with higher nivolumab response and lower anti-PD1 resistance compared to other subtypes of TME (20). Therefore, there may be benefit in incorporating the characterization of TME in future clinical investigations as a prognostic factor for immunotherapy response.

Currently the standard first-line treatment for RMNPC is gemcitabine/cisplatin chemotherapy, associated with a median OS of 29.1 months in a RCT (7). Sequential mono-chemotherapy has typically been used after disease progression despite this regimen, but is not reliably effective. A small uncontrolled trial demonstrated activity and safety of pembrolizumab as second-line therapy for RMNPC that progressed on platinum chemotherapy (21). In a meeting presentation, Chan et al reported results of a phase III RCT studying pembrolizumab compared to monochemotherapy in RMNPC patients progressing despite prior platinum-based chemotherapy. There was similar activity (objective response rate 21.4% and disease control rate 50.4%) and less toxicity, but no improvement in OS (HR 0.90 [95%CI, 0.67-1.19]) compared to mono-chemotherapy (22). We await the final report of this RCT, as this result is puzzling. In another phase II RCT, spartalizumab (PD-1 inhibitor) compared to chemotherapy had lower median PFS (1.6 months, P=0.915), longer OS (25.2 months, 95% CI, 13.1–not estimable) and longer duration of response (10.2 months, 95% CI, 7.4–NE) in patients with NPC who progressed on or after platinum-based chemotherapy (23). Common immune-mediated toxicities and other side effects of PD-1 inhibitors reported in these trials include hypothyroidism, rash, pruritis, cough, fatigue, and constipation, which were not reported in our case (22, 23). In RCTs studying patients with incurable recurrent or metastatic non-nasopharyngeal squamous cell carcinoma of the head and neck progressing despite platinum-based chemotherapy, Ferris et al (progression within 6 months) and Cohen et al (progression within 3-6 months) reported improved OS (HR: 0.70; 97.73% CI, 0.51 to 0.96; p=0.01; and HR: 0.80; 95% CI, 0.65–0.98; p=0.0161) with nivolumab and pembrolizumab, respectively, compared to mono-chemotherapy or cetuximab (8, 24). Regarding treatment-naïve RMNPC, three RCTs have reported PFS improvement with the addition of PD-1 inhibitors (toripilamab, camrelizumab, and tislelizumab) to first-line gemcitabine/platinum for RMNPC (25–27).

Four years from his diagnosis of widespread metastatic disease, our patient is essentially living a completely normal life due to an exceptionally deep and durable radiological and serological response to PD-1 inhibitor therapy. We doubt this would have been achieved with palliative mono-chemotherapy, and believe this report provides support for the use of PD-1 inhibitors in selected patients with incurable disease. However, RCTs suggest that the optimal approach may be to add these agents to standard therapy in the first-line RMNPC setting (25–27). Continued research to identify the optimal sequence and methods for stratifying therapies for individual patients with metastatic NPC is needed.

## Data availability statement

The data analyzed in this study is subject to the following licenses/restrictions: Data for this case report were obtained from patient charts that are found in the Powerchart system of London Health Sciences Centre, which are only accessible to students and staffs. Requests to access these datasets should be directed to https://www.sjhc.london.on.ca/medical-affairs.

## Ethics statement

The studies involving humans were approved by Western Health Sciences Research Ethics Board. The studies were conducted in accordance with the local legislation and institutional requirements. Written informed consent for participation was not required from the participants or the participants' legal guardians/next of kin in accordance with the national legislation and institutional requirements. Written informed consent was obtained from the minor(s)' legal guardian/next of kin for the publication of any potentially identifiable images or data included in this article.

## Author contributions

WW completed data collection and wrote the manuscript. BP and EW reviewed and edited all versions of the manuscript. EW served as the medical oncologist for the case and overlooked the project as the corresponding author. NR, PG, and KK were the radiation oncologist, pediatric oncologist, and pathologist, respectively, for the case and all contributed to reviewing the manuscript. All authors contributed to the article and approved the submitted version.
